# Formations in the Pulp-Cavity

**Published:** 1890-12

**Authors:** Wm. P. Cooke


### International Dental Journal.

VOL. XI. DECEMBER, 1890. NO. 12.

### Original Communications.[1]

### FORMATIONS IN THE PULP-CAVITY.[2]

BY WM. P. COOKE, D.M.D.

THESE deposits have long been recognized. In 1780 and in 1835 the fact was spoken of; also by Tomes in 1846.

These formations may be classified as follows:

1. Secondary dentine,—normal.
2. Nodules attached to the wall of the pulp-cavity.
3. Nodules loose in the pulp cavity.
4. Calcifications, loose, easily distinguished from the others by their white chalky appearance.

The fact that dentine is deposited by the pulp as the teeth are worn down was recognized by Dr. John Hunter in 1800. The tubuli are continuous with the normal dentine, and have a similarity of arrangement in the dentine of repair, but in the loose and nodular formation there is no similarity of direction.

*Cause.*—The cause may be external or spontaneous. They were believed by Mr. Hulme to be caused by irritation set up by a decaying cavity, the deposit forming as long as the irritation is kept up.

---

[1] The editor and publishers are not responsible for the views of authors of papers published in this department, nor for any claim to novelty, or otherwise, that may be made by them. No papers will be received for this department that have appeared in any other journal published in the country.

[2] Read before the American Academy of Dental Science, Boston, Mass., May 7, 1890.

The cause may be similar to the one which causes ligament to be changed to cartilage, cartilage to bone, the deposit in the coats of the arteries, the ossification of the cartilage of the ribs, and the deposits in the brain. Some may be caused by age; for others we rely upon a constitutional tendency. Syphilis and gout cause nodes in bones and possibly these formations in the teeth. When these are laid down there is an increased supply of blood, so any cause which produces this increase of blood-supply may favor the deposit, such as caries, recessions of the gums, or absorption of the alveolar process, by which means the pulp is more exposed to heat and cold; large metallic fillings, lack of occluding teeth, undue striking and irritation by clasps. Wedl thinks, while many old teeth have new formations, as all do not have them, the cause must be sought in an independent process which is not accompanied by pain. Chronic pericementitis may be a cause,—if on one root only, the deposit will be laid down in that root.

*Effects.*—The effect of these deposits is not wholly known. In some cases severe neuralgia is traceable to this source. In these cases the patient may have sharp lancinating pains, teeth very sensitive to thermal changes, also to excavation; itching and slight uneasiness at any time, a continuous boring pain in one spot, sensitiveness of enamel on scratching with finger-nails, slight external inflammation of gum, and soreness of the tooth to percussion. Wedl thinks that pain is not produced by calcifications until they are quite large. Cases are on record where several teeth have been removed at different times for the same patient, and the deposit found in each case was supposed to be the cause of the neuralgia. If in these cases we must extract, it is advised to first allay the pain, as the neuralgia is less liable to follow.

These deposits may have an important bearing in pulp-capping. Some writers claim that when the layer of odontoblasts have been destroyed, no new deposits of dentine will take place, but that we may expect that in a healthy pulp calcification will occur at the point of exposure.

*Shape and location.*—These deposits vary from a small speck, scarcely visible without a glass, to those that nearly fill the pulp-cavity. These are surrounded by a delicate membrane, which prevents their uniting with the wall of the pulp-chamber. This accounts for the difficulty we have in devitalizing such teeth, the application acting on a small portion of the membrane only. A touch upon the deposit causes pain by producing pressure on the live tissue on the other side of the deposit.

These calcifications are found in the bundles of connecting tissue and the sheaths of the blood-vessels.

Salter thinks the calcifications begin at different points, and at last pass into one mass. He also thinks the new deposit is at last fused and confounded with the dentine. If this were so, we would find the whole pulp-cavity filled, which we rarely, if ever, do. Many times the deposit is found at the mouth of the pulp-canal, and must be removed before the canal can be cleaned. When the deposit is loose this is comparatively easy, but when the canal is covered by one of these growths that comes from the wall of the pulp-chamber, especially from the point of bifurcation of the roots, it is not very clear which way we must cut to reach the canal. A case in point, where the deposit was drilled three-fourths through and then the pulp-chamber and remaining roots were filled, after-trouble caused the removal of the tooth, and this might have been avoided by drilling a little farther, but caution forbade such a course. The largest deposit I have found in the molar teeth was in that class that have probably stood the shock of mastication for years; also in the dark-yellow teeth, many of which have been lost by Riggs's disease. With a little experience we may select from a number of teeth a majority which will have deposits in them. This refers to the dark-yellow dentine deposits, not to the calcification which appears to be in much younger teeth.

In one case of a lower wisdom tooth, where probably the decay had been rapid, and had progressed till the pulp was exposed, the whole of the pulp-chamber was filled with a white deposit, which was loose and had a membrane between it and the wall.

In the canines the apex of the pulp is most often ossified, but in many we find the small round nodules, which are in the substance of the pulp. Several may be present. They are deposited directly under a filling and seemed to be caused by it, though they may have been there before it was inserted.

The safe course in the treatment of devitalized teeth is to open well into the pulp-chamber, and by this means we may obtain access into the canals. In the majority of cases I think no trouble arises primarily from these deposits.

*Frequency.*—Salter claimed that every tooth had its pulp more or less ossified, and that it occurs oftener in the roots. Some claim it is more frequent in young persons, and in deciduous more than in permanent teeth.

I do not find this to be the case, but rather the reverse. It undoubtedly occurs more with chronic than with acute caries.

These deposits are not confined to humanity, as similar ones are found in the teeth of the deer, hare, pig, and walrus.

These deposits have no relation to the exostosed teeth. In the *Dental Cosmos*, vol. v. (1863–64) is the record of ten thousand teeth examined for exostosis, with the following results:

| | Per cent. |
|---|---|
| Carious teeth, 6200, exostosed 319 | 5 |
| Sound teeth, 3000, exostosed 85 | 4 |
| Average exostosed | 4 |
| First, molar, in 4000, 210 exostosed | 5 |
| Second, bicuspids, in 2000, 94 exostosed | 4 |
| Third, incisors and canines, 250, 10 exostosed | 4 |

The greatest frequency is found in molars. Exostosis being caused by a constant, though slight, irritation to the pulp, and in this respect these deposits resemble it.

Some five years ago I commenced a series of investigations to determine the frequency of these deposits in human teeth. I have examined teeth collected in three different parts of the State and with the same results. The teeth, being dry, were cracked upon a small anvil and the contents of the pulp-chamber examined by a strong glass. Accurate records were kept as to soundness, decay, etc., and with the following results:

| | Teeth examined. | Deposit. | Per cent. |
|---|---|---|---|
| Incisors decayed | 521 | 28 | 5 |
| " not decayed | 179 | 11 | 5 |
| | 700 | 39 | 5 |
| Bicuspids decayed | 539 | 29 | 5 |
| " not decayed | 161 | 7 | 4 |
| | 700 | 36 | 5 |
| Cuspids decayed | 140 | 10 | 9 |
| " not decayed | 160 | 31 | 19 |
| | 300 | 41 | 13 |
| Superior molars decayed | 1054 | 293 | 27 |
| " " not decayed | 551 | 191 | 34 |
| | 1605 | 484 | 30 |
| Inferior molars decayed | 1089 | 297 | 27 |
| " " not decayed | 606 | 145 | 23 |
| | 1695 | 442 | 26 |

RECAPITULATION.

| | Total examined. | Deposit. | Average per cent. |
|---|---|---|---|
| Molars | 3300 | 926 | 28 |
| Incisors | 700 | 39 | 5 |
| Bicuspids | 700 | 36 | 5 |
| Cuspids | 300 | 41 | 13 |
| | 5000 | 1042 | |

